# Inhibition of cyclophilins alters lipid trafficking and blocks hepatitis C virus secretion

**DOI:** 10.1186/1743-422X-8-329

**Published:** 2011-06-28

**Authors:** Leah J Anderson, Kai Lin, Teresa Compton, Brigitte Wiedmann

**Affiliations:** 1 Novartis Institutes for Biomedical Research, Inc Cambridge, Massachusetts 02139, USA

## Abstract

Host cyclophilin (cyp) inhibitors, such as NIM811, efficiently inhibit replication of hepatitis C virus (HCV) and have shown significant promise in recent clinical trials for the treatment of chronic HCV. It is therefore important to fully understand the mechanism of action of these therapeutic agents. Data obtained from comprehensive systems biology approaches have led to the hypothesis that the antiviral activity of cyclophilin inhibitors is mediated through impairing the cellular machinery on which HCV relies to traffic cofactors necessary for formation of the replication complex. Indeed, our results demonstrate when cyclophilins are inhibited by NIM811, lipid and protein trafficking within the VLDL pathway is impaired. Following treatment of replicon or HCV infected cells with NIM811, intracellular lipid droplets (LD) more than double in size and decrease in number. Changes in the LDs in response to cyclophilin inhibition are dependent upon expression of viral proteins. Additionally, in cells treated with NIM811, apoB accumulates in a crescent or ring shaped structure surrounding the enlarged LDs and is no longer secreted. Silencing of cypA or cyp40 using siRNA had a similar effect on LD size and apoB localization as compound treatment, suggesting these cyclophilins may play an important role in lipid and apoB trafficking. Interestingly, the decrease in apoB secretion correlates with a decrease in release of viral particles in HCV infected cells. Altogether, these results add a new level of complexity to the mechanism of action of cyclophilin inhibition, and suggest the role for cyclophilins in the virus life cycle extends beyond replication to virus release.

## Introduction

Chronic Hepatitis C virus (HCV) infection, a major cause of chronic hepatitis, cirrhosis and hepatocellular carcinoma, afflicts approximately 3% of the world's population [1]. The current standard of care is pegylated interferon and ribavirin, which shows poor tolerability and is only capable of achieving a sustained viral response in half of genotype 1 infected patients [2]. Although new direct acting anti-virals (DAA) are on the immediate horizon for treatment of HCV patients in the clinic, the risk for resistance mutations arising in response to these drugs necessitates continued development of new therapeutic agents [3]. Cyclophilin inhibitors, such as NIM811 and alisporivir, target host cell proteins and have the capacity to increase the barrier to resistance when used in combination with DAAs *in vitro *[4-6]. As this class of compounds continues to be investigated in the clinic with much success, it is important to have a more thorough understanding of their mechanism of action [7].

HCV is a small enveloped virus with a positive, single strand RNA genome of ~9.6 kb that encodes a single polyprotein. The polyprotein undergoes co- and post-translational processing into 10 viral proteins, which form the replication complex in association with the membranous web. The membranous web consists of viral proteins, replicating viral RNA and altered cellular membranes from the endoplasmic reticulum (ER). Formation of the membranous web is thought to protect the replication complex from host innate defense proteins so replication can proceed [8]. HCV replication is tightly linked to lipid biology, and the necessity of fatty acids and cholesterol for construction and maintenance of a membranous web has been demonstrated [9-12]. In the cell, the main source of neutral lipids is the lipid droplet (LD), where they are stored in the form of triacylglycerols (TAG) and cholesterol esters. When needed, these neutral lipids are mobilized from the LD to the ER and serve as substrates for lipid metabolism, membrane synthesis and steroid synthesis [13].

Many cellular proteins have been identified to play a role in the life cycle for HCV, but perhaps the most recognized of these are the cyclophilins (cyp). Cyclophilins are peptidyl-prolyl isomerases that assist in protein folding by catalyzing the cis/trans isomerization of the peptidyl-prolyl bond [14]. In addition to their enzymatic activity, other functions attributed to the cyclophilins include mediating cholesterol transport, acting as protein chaperones, and RNA splicing [15]. In the case of HCV, many cyps have been deemed important for viral replication and possibly virus assembly [16-20]. CypA emerges from these studies as the predominant cyclophilin, and is thought to not only assist in viral protein folding, but also to enhance interactions between viral proteins and RNA, polyprotein processing and replication complex formation [7]. In our hands, we find cypA, cyp40 and cypH to be important for HCV replication [17]. While the biological function of cyp40 is not completely characterized, this co-chaperone can be found in complex with heat shock proteins (hsp90/hsp70) and cholesterol [21].

Following the discovery that cyclosporin A (CsA) has anti-viral activity when combined with interferon in HCV patients, several non-immunosuppresive CsA analogs, including NIM811 and alisporivir, have been intensely investigated as a possible new therapy for chronic HCV. NIM811 binds to cyclophilins with a greater affinity than CsA, and this binding affinity correlates with anti-viral activity in the HCV replicon assay [4]. NIM811 is a powerful tool that allows the exploration of the relationship between cyclophilin biology and the virus life cycle. In pull down experiments using immobilized compound, we found NIM811 binds to proteins involved in intracellular trafficking [17]. Similarly, an siRNA screen in HCV replicon cells identified that proteins potentially involved in virus replication were also involved in protein and lipid trafficking [17]. Taken together, these results have led to the hypothesis that NIM811 may inhibit viral replication by impairing the cellular machinery on which HCV relies upon to traffic cofactors necessary for formation of an active replication complex. Our results demonstrate that in addition to altering lipid trafficking, as evidenced by an accumulation of neutral lipids in LDs, cyp inhibition also results in decreased apoB secretion through the VLDL pathway. We find that cyclophilins are also important for the secretion HCV, as HCV virion assembly and release is highly dependent upon the VLDL pathway and can be blocked by treatment with NIM811 [22]. Correspondingly, siRNAs specific to cypA or cyp40 in replicon cells have the same effect as NIM811 on the LD size and apoB transport. These results suggest that NIM811 mediated inhibition of cypA and cyp40 are responsible for the changes in protein and lipid trafficking and likely play a role in secretion of HCV.

## Results

**Figure 1:**
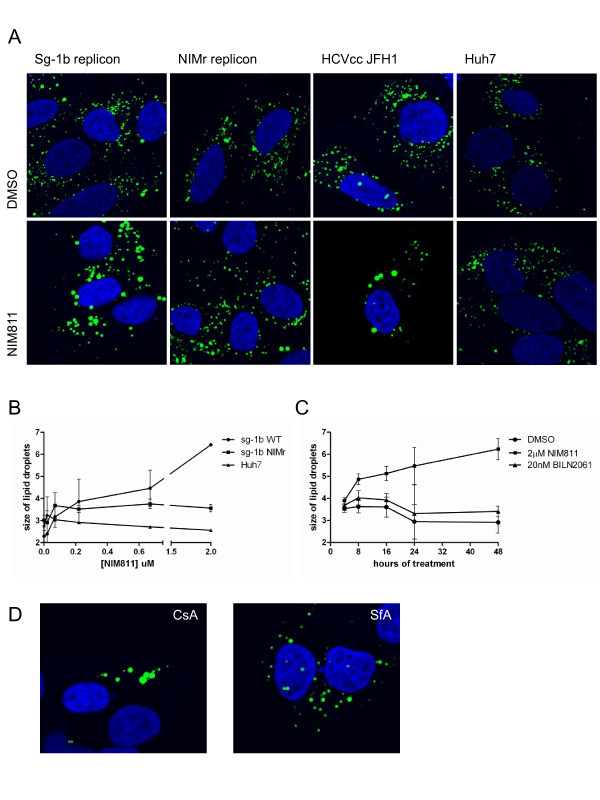
***HCV replicon cells treated with NIM811 have enlarged lipid droplets***. Wild type sub-genomic 1b (sg-1b) replicon, a sg-1b NIMr replicon, Huh7.5 cells infected with JFH1 HCVcc or Huh7 naïve cells were grown in the presence of NIM811 or DMSO vehicle control. Following compound treatment, cells were fixed, permeabilized and stained for lipid droplets using BODIPY 493/503. A representative image of the lipid droplets in cells treated with 2μM NIM811 (10X EC_50_) for 24h is shown (A). B) sg-1b, sg-1b NIMr and naïve Huh7 cells were treated with increasing concentrations of NIM811 for 48h. Images of lipid droplets were acquired and quantified by the Cellomics ArrayScan VTI HCS reader. The object area of lipid droplets in each cell type averaged from three experiments is graphed ± standard deviation. C) sg-1b replicon cells were treated with indicated compounds for 48h and the size of the lipid droplets quantified as in B. The object area of the lipid droplets are graphed ± standard deviation. BILN2061 is an NS3 protease inhibitor with a replicon EC_50 _of 2nM. D) sg-1b replicon cells were grown in the presence of cyclosporin A (CsA, 8μM) or sanglifehrin A (SfA, 10μM) for 24hr. Lipid droplets were fixed and stained as in (A). A representative image is shown, where green represents lipid droplets and blue represents the nuclei (DAPI).

In light of our previous findings, we became interested in understanding the interplay between cyclophilins, HCV and lipid metabolism. The morphology of lipid droplets is often indicative of the metabolic state of a cell [23,24], so we began by investigating whether cyclophilin inhibition has any affect on lipid droplet morphology. Following 24h treatment with 2μM NIM811 (10X EC_50_), the lipid droplets were stained by BODIPY 494/503 in sg-1b replicon cells as well as JFH1 HCVcc infected cells. We observed that the LDs were significantly larger and decreased in number (top and bottom panels, Figure 1A) following treatment with compound. In contrast, the lipid droplets in a sg-1b NIM811 resistant (NIMr) replicon cell line and in Huh7 naïve cells remained as small punctate dots in the cytoplasm (second and fourth panels, Figure 1A) [5]. We confirmed that BODIPY 493/503 was staining LDs by using an antibody specific for ADRP (adipocyte differentiation related protein), a lipid droplet resident protein [13] (data not shown). Quantification of lipid droplet size over a range of NIM811 concentrations showed that while the lipid droplets in sg-1b replicon cells double in size in the presence of NIM811, the size of lipid droplets remained constant in both sg-1b NIMr replicon cells as well as naïve Huh7 cells (Figure 1B). These results indicate the expression of wild type non-structural viral proteins is necessary to observe changes in LD morphology. A time course analysis of lipid droplets in the presence of NIM811 demonstrated that lipid droplets begin to increase in size within the first 8 hours of compound treatment and become twice as large between 24h and 48h, whereas the NS3 protease inhibitor BILN2061 did not alter the size of lipid droplets (Figure 1C). Similarly, other cyclophilin inhibitors tested, CsA and sanglifehrin A (SfA), resulted in significantly larger lipid droplets in sg-1b replicon cells but not naïve Huh7 cells, confirming this phenotype is specific to cyclophilin inhibition in the presence of HCV proteins (Figure 1D).

**Figure 2:**
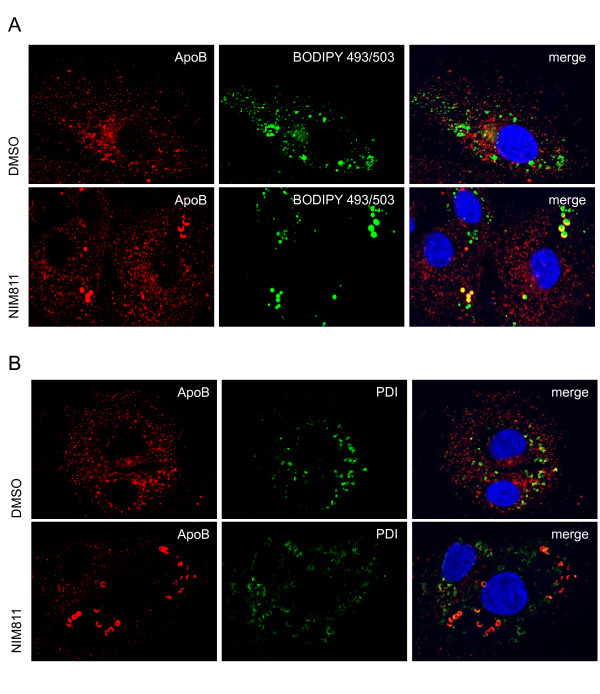
***ApoB accumulates as crescents in the ER surrounding the lipid droplets of sg-1b replicon cells treated with NIM811***. Sg-1b replicon cells were grown in the presence of compound for 24h, after which they were fixed, permeabilized and stained using antibodies specific for apoB, NS5A and PDI. BODIPY 493/503 was used to visualize lipid droplets, and DAPI for nuclei. A) ApoB (red) is dispersed throughout the secretory pathway in sg-1b cells grown in the presence of DMSO. Following growth in NIM811, apoB forms ring-like and crescent shaped structures surrounding the lipid droplets (green). B) In DMSO treated cells, some of the apoB protein in replicon cells is localized to the ER, as shown by the overlapping staining pattern with the ER marker PDI. The ring-like and crescent shaped structures containing most of the detectable apoB protein co-localize with PDI in the presence of NIM811.

**Figure 3:**
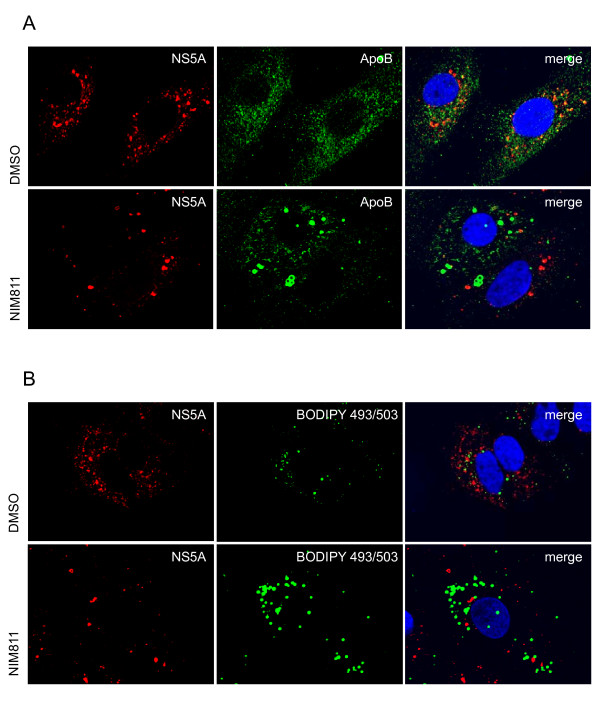
***NS5A does not co-localize with apoB crescents in sg-1b replicon cells treated with NIM811***. Sg-1b replicon cells were treated and stained as in Figure 2. A) NS5A (red) localizes to regions that also contain apoB within replicon cells treated with DMSO, whereas NS5A and apoB no longer co-localize in the presence of NIM811. B) NS5A and lipid droplets are in close apposition, but the two do not co-localize in sg-1b replicon cells regardless of compound treatment.

Lipid droplets are the primary source of TAGs for VLDL synthesis in hepatocytes [25]. VLDL particles are formed in the liver through the interaction of apoB with lipids including cholesterol, phospholipids and TAGs [26]. To determine whether the alterations in lipid droplets resulting from cyclophilin inhibition affected the VLDL pathway, sg-1b replicon cells were treated with NIM811 or DMSO vehicle control for 24h and stained for apoB, the major protein component of VLDL [27]. Figure 2A and 2B show that in DMSO treated cells, apoB is distributed throughout the cell and in the ER, as expected. Following treatment with NIM811, apoB accumulates in ring-like structures around the enlarged lipid droplets, similar to the apoB crescents described previously [28]. The apoB crescents partially co-localize with PDI, a protein that is retained in the ER by its KDEL sequence [26], indicating that the enlarged lipid droplets are either within the ER or in very close proximity with the ER membrane (Figure 2B). Although NS5A was found to co-localize with some of the apoB in DMSO treated replicon cells, as shown previously [22], apoB in NIM811 treated cells no longer localizes with NS5A (Figure 3A). We see the same staining pattern when we use an antibody to NS3 (data not shown). These results further support that trafficking of apoB in replicon cells is interrupted when cyclophilins are inhibited. Although NS5A can be found close to lipid droplets, we are unable to detect any co-localization of NS5A with the lipid droplet in the sub-genomic replicon cell system (Figure 3B). The small accumulations of NS5A found in NIM811 treated replicon cells co-localize with calnexin (data not shown) and likely reflect unfolded protein resulting from cyclophilin inhibition [18]. Similar results to those shown in Figures 2 and 3 were obtained with JFH1 HCVcc infected cells (data not shown).

**Figure 4:**
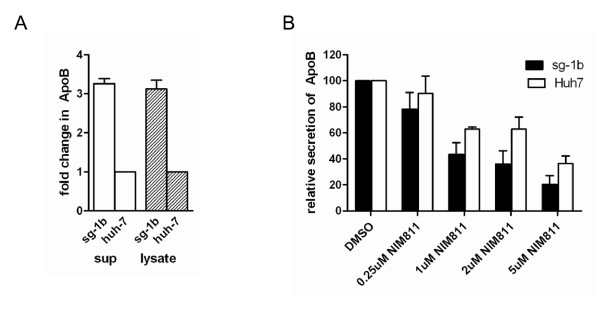
***The effect of viral proteins and cyclophilin inhibition on apoB secretion***. A) Supernatants and whole cell lysates from equal numbers of sg-1b replicon and naïve Huh7 cells were used to quantify apoB protein by ELISA. The average apoB protein levels from two experiments was normalized to naïve Huh7 cells and graphed as fold change in apoB protein. B) Sg-1b replicon and Huh7 naïve cells were treated with varying concentrations of NIM811 for 48h. ApoB was measured in the supernatants by ELISA. The amount of apoB protein was normalized to DMSO controls for each cell type and plotted as relative secretion ± standard deviation from three experiments.

To further understand the data suggesting viral protein expression is necessary for cyclophilin inhibition to result in the enlarged lipid droplet phenotype and apoB crescent formations, we next compared apoB expression and secretion in sg-1b replicon cells and naïve Huh7 cells. Interestingly, there is a ~3-fold increase in apoB in the supernatant from Huh7 cells containing viral proteins (sg-1b) compared with naïve Huh7 cells (Figure 4A). There is also a 3.1 fold increase in apoB in whole cell lysates (representing intracellular apoB) from sg-1b cells compared with Huh7 cells. To determine whether the formation of apoB crescents in NIM811 treated cells affected apoB secretion, we measured the amount of apoB in the supernatants of sg-1b replicon cells and naïve Huh7 cells by ELISA. As shown in Figure 4B, we observed a dose dependent decrease in the amount of apoB in the supernatant following 48h treatment with NIM811. The effect of cyclophilin inhibition on apoB secretion is more pronounced in sg-1b cells expressing viral proteins compared with naïve Huh7 cells, where we consistently observe an ~20% difference at each concentration (p < 0.01 comparing secretion from sg-1b and naïve Huh7 cells at each concentration by paired students t-test). These results confirm that secretion of apoB is impaired when cyclophilins are inhibited by NIM811.

**Figure 5:**
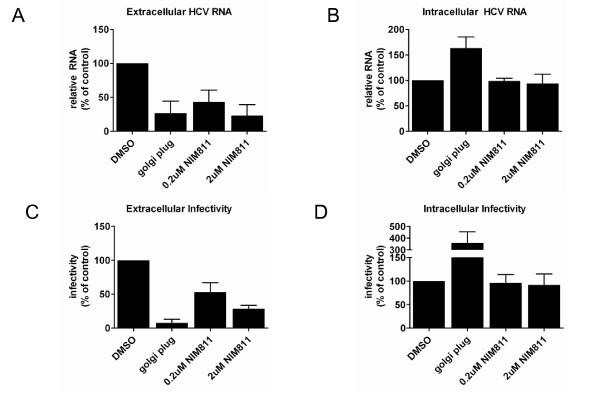
***NIM811 blocks the release of HCV RNA and infectious virions from infected cells***. Huh7.5 cells were infected with JFH1 HCVcc for three days. Following infection, cells were washed and treated with the indicated compounds for 8h. Intracellular and extracellular virus was harvested as described in materials and methods, and assayed for viral RNA content by qRT-PCR (A and B) or infectivity (C and D). GolgiPlug prevents secretion of infectious particles, resulting in an accumulation of intracellular viral RNA and infectious particles. There is a dose dependent decrease in viral RNA and infectious particles in the media of infected cells treated with NIM811.

The assembly and release of infectious HCV particles is tightly associated with the VLDL secretory pathway [22,29]. Given our results demonstrating that NIM811 inhibits the secretion of apoB from replicon and HCVcc infected cells, we hypothesized that NIM811 could also block the release of infectious particles. To test this hypothesis, we examined viral RNA and infectious particle release in the supernatant of infected cells 8h post-compound treatment to minimize the effect of compound on replication. It was shown previously that 8h is sufficient to detect a change in particle release using GolgiPlug (brefeldin A) [29]. Huh7.5 cells were infected with JFH1 HCVcc and incubated for three days, which resulted in >80% HCV positive cells (data not shown). We used GolgiPlug as a control, as this compound inhibits intracellular transport and has been previously demonstrated to inhibit HCV release from infected cells without affecting virus replication [29]. As expected, GolgiPlug resulted in an accumulation of HCV RNA and infectious particles. The addition of 0.2μM NIM811 (~EC_50_, data not shown) to infected cells resulted in a 50% decrease in the release of both HCV RNA and infectious particles into the supernatant compared with DMSO control (Figure 5A and 5C). The release is inhibited further by addition of 2μM NIM811, which resulted in a 75% decrease. Analysis of the intracellular content reveals that the amount of HCV RNA and infectious particles in NIM811 treated samples is equivalent to the DMSO control (Figure 5B and 5D). In contrast to GolgiPlug treated cells, there is a lack of accumulation of HCV RNA or infectious particles in NIM811 treated cells, which can be explained by the effect of NIM811 on replication. These results suggest that cyclophilins are not only important for replication; they also play a potential role in the assembly/egress process for HCV.

**Figure 6:**
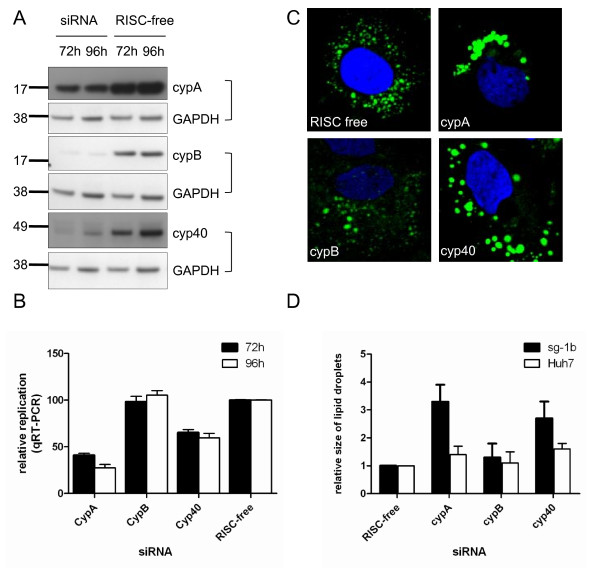
***Enlarged lipid droplets correlate with inhibition of HCV replication using siRNA knockdown***. A) sg-1b replicon cells were transiently transfected with siRNA to target cypA, cypB and cyp40. A RISC-free siRNA construct was used as a control. Whole cell lysates were analyzed for target protein expression by Western blot at 72h and 96h post-transfection. B) Total RNA from siRNA transfected cells was extracted and used for quantitative real-time RT-PCR to measure copies of HCV. Replication in RISC-free control cells was set to 100% for each time point. Results are the average of 4 independent experiments, ± standard deviation. CypA and cyp40 siRNA result in a statistically significant decrease in replicon activity, p < 0.01 for cypA and p < 0.05 for cyp40 compared to RISC-free siRNA by Students paired t-test. C) At 96h post-transfection, lipid droplets were visualized as described. Representative images for each siRNA are shown. D) 20X images were acquired and quantified by the Cellomics ArrayScan VTI HCS reader. The mean fluorescence intensities for BODIPY 493/503 were normalized to the RISC free siRNA control. Those siRNA constructs that resulted in inhibition of HCV [17] also caused the lipid droplets to become enlarged, similar to what is seen with NIM811 treatment.

**Figure 7:**
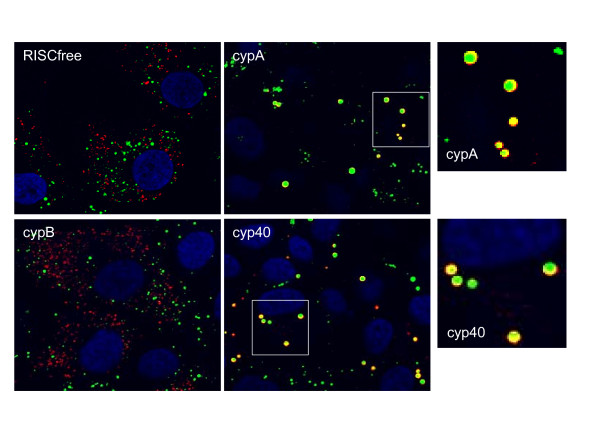
***ApoB crescents form in replicon cells transfected with siRNA to cypA and cyp40, but not cypB***. Sg-1b cells were transfected with siRNA as in Figure 6. At 96h post-transfection, cells were fixed, permeabilized and stained for lipid droplets (green) and apoB (red) as described. Representative images are shown.

To gain insight into the relationship between cyclophilins and lipid droplets, we used siRNA to knock down cyclophilin protein expression in sg-1b replicon cells and examined the lipid droplets. Western blot analysis (Figure 6A) demonstrates that siRNAs targeting cypA, cypB and cyp40 result in a significant reduction in protein expression. Although cypA is still abundant in the cell following siRNA transfection, this level of protein reduction was sufficient to reduce replication by 60% at 72h and 80% by 96h (Figure 6B). A modest, but statistically significant, decrease in replication is seen with siRNA to cyp40. These results are similar to those previously published using a sg-1b replicon expressing luciferase [17]. Interestingly, inhibition of replicon activity by siRNA correlates with an increase in lipid droplet size (Figure 6C). Compared with the RISC free siRNA control, cypA and cyp40 siRNAs result in an approximately 3-fold increase in lipid droplet size in sg-1b replicon cells, as quantified by high content imaging (Figure 6D). In contrast, cypB siRNA does not affect the size of lipid droplets. Interestingly, there is no difference in the size of lipid droplets when naïve Huh7 cells are transfected with cyclophilin siRNAs (Figure 6D), further supporting the data from experiments using NIM811 treatment. As shown in Figure 7, apoB crescents accompany the enlarged lipid droplets that form in sg-1b replicon cells transfected with siRNA to cypA or cyp40, but not cypB. These data confirm that the increased lipid droplet size and decreased apoB secretion upon NIM811 treatment is mediated through inhibition of cypA and cyp40.

## Discussion

The current standard of care for chronically infected HCV patients lacks efficacy and does not achieve a sustained viral response in all patients, especially those infected with genotypes 1 and 4 [2]. While promising, the new DAAs coming into the clinic present the additional challenge of resistance mutations arising, thus necessitating additional approaches [3]. By targeting a cellular factor, cyclophilin inhibitors have the potential to increase the barrier of resistance as well as provide broad genotype coverage [4,7]. Cyclophilins are best known for their role as cellular chaperones in protein folding and trafficking, and many of the HCV non-structural proteins have been identified as substrates [7]. As cyclophilins appear to be important in the life cycle of a number of viruses, including HIV, human papillomavirus (HPV), cytomegalovirus (CMV), and vesicular stomatitis virus (VSV), it is of utmost importance to understand the biology of these proteins and their relationship with viruses. Here, we use NIM811 as a tool cyclophilin inhibitor and find that in addition to viral replication, cypA and cyp40 are important for the release of infectious HCV particles.

In Huh7 cells expressing viral proteins, either from the replicon or JFH1 HCVcc, NIM811 treatment results in enlarged lipid droplets and apoB crescent formation. These changes correlate with what is seen in replicon cells transfected with siRNA to cypA or cyp40, indicating that the effect seen with NIM811 is due to inhibition of cypA and/or cyp40. In contrast, we were unable to detect any change in the lipid droplets or apoB trafficking in Huh7 cells. Altogether, these results lead to the question: why does NIM811 induce such dramatic changes only in the presence of viral proteins?

Hepatocytes are responsible for a large proportion of cholesterol and lipid synthesis, which is assembled into VLDL particles along with apoB, allowing the lipids to be transported in the bloodstream and used as an energy source. The life cycle of HCV is tightly associated with lipid metabolism, including the secretory pathway for VLDL, and the availability of lipids (fatty acids, cholesterol and sphingolipids) directly influences the rate of HCV replication [9,17]. It is therefore not surprising that replication of the virus is restricted to this host cell type [9,11,12,30,31]. Core and NS5A are thought to induce changes in the expression of genes which are involved in lipid metabolism and cholesterol regulation [10,31]. These changes create a lipid rich environment which favorably supports the formation of the membranous web as well as assembly, while inadvertently leading to steatosis [10,31,32]. Expression of NS4B alone can induce formation of the membranous web independent of any other viral genes, and it has recently been demonstrated to stimulate signaling pathways that result in accumulation of lipids [33,34]. Altogether, these data indicate that in cells expressing viral proteins, there is an overall up-regulation of lipid synthesis. Taken with our observations that the effect of NIM811 is more pronounced in cells expressing viral proteins than in naïve Huh7 cells, and apoB expression and secretion is upregulated in sg-1b replicon cells, we hypothesize that NIM811 is blocking pathways stimulated by viral proteins, resulting in an accumulation of excess lipids and apoB.

TAG stored in LDs is transferred to the ER lumen to interact with apoB and form pre-VLDL particles [26]. Many chaperones are known to assist in this process, including cyclophilin B [35]. By siRNA knockdown, we demonstrate that cypB does not have a role in the alterations in lipid droplets and apoB crescent formation, whereas cypA and cyp40 do. One explanation may be that cypB protein is incompletely eliminated by siRNA transfection, and the small amount of remaining protein is sufficient to accomplish this function. A more intriguing explanation is independent of cypB, and involves a role for cypA and cyp40 in delivering cholesterol and TAG from the lipid droplet to the ER to form pre-VLDL particles with apoB. A complex of cypA, cyp40, caveolin and Hsp56 form a chaperone complex which transports cholesterol within the cytoplasm. In the presence of CsA, this interaction is disrupted and transport of cholesterol is interrupted [36]. Caveolin has been shown to be targeted to the core of LDs in addition to other membranes within the cell, making it likely that the caveolin/cypA/cyp40 complex can shuttle cholesterol between the LD and ER [37]. ApoB crescent formation increases when cells are treated with inhibitors of cholesterol synthesis, and if cholesterol is not available for VLDL synthesis, the secretion of apoB is impaired [26]. Thus, the possibility exists that NIM811, like CsA, disrupts the interaction of cholesterol with cypA and cyp40, thereby preventing cholesterol from leaving the LD and trafficking to the ER, where it would interact with apoB to form pre-VLDL. Binding of NIM811 to cypA and cyp40 and the subsequent block in caveolin/chaperone/cholesterol complex formation at the LD would therefore result in a lack of cholesterol trafficking into the ER for VLDL synthesis and subsequent apoB crescent formation. This explanation provides for the enlarged lipid droplets that are seen upon treatment with NIM811 as well as the accumulation of apoB that is seemingly stuck in the ER.

Perhaps the most interesting finding presented here is NIM811 inhibits the egress of virus particles from JFH1 HCVcc infected cells. The mechanism whereby cyclophilin inhibition may target this process is at least two tiered. There is mounting evidence that NS2 plays a central role in the assembly and production of infectious particles, through interactions with various viral proteins including the envelope proteins, p7, NS3 and NS5A [38-43]. In a recent report, NS2 has also been added to the list of viral proteins that may require cypA for proper folding [44]. In support of this notion, it was found that full length genomes are more sensitive to inhibition by cyclophilin inhibitors, and this increased sensitivity is dependent upon the presence of NS2 [44]. If NS2 is indeed crucial for the production of virus, and cypA is important for NS2 function, NIM811 inhibition of virus secretion may be at the level of NS2 protein folding mediated by cypA. While this explanation takes into account the activity of NIM811 in HCVcc infected cells, NIM811 affects apoB and the LDs in sub-genomic replicon cells, suggesting that other viral proteins are also involved in this process.

The second tier of NIM811 mediated inhibition of secretion is by disrupting the intricate relationship between VLDL assembly and HCV particle assembly [29,45-47]. VLDL assembly can be delineated into two steps, which are separated by the cellular compartment in which they occur. In the first step, microsomal triglyceride transfer protein (MTP) transfers triglycerides from the LD or the ER to apoB in the lumen of the ER. The second stage of assembly is thought to occur in the ER/golgi luminal compartment, where additional triglycerides are added to the complex, thereby decreasing its density. Both apoB and apoE are important structural components of VLDL [48]. HCV circulates in the plasma of infected patients in a complex with VLDL, and agents that inhibit the assembly of VLDL block the secretion of HCV [22]. In addition, both apoB and apoE have been shown to be important host factors in the assembly and/or egress stage of the HCV life cycle, altogether indicating that HCV hijacks the VLDL assembly pathway for assembly and egress of viral particles. While it is increasingly clear that HCV assembly and release is dependent upon the VLDL machinery, the exact details of where the virus enters the pathway have not been elucidated [29,45-47]. NIM811 appears to block VLDL assembly at the stage where lipids would be transferred from the LD, which is closely associated with the ER, to apoB in the ER membrane, preventing apoB from engaging the secretory pathway. It is tempting to speculate that this is also where assembled viral particles may become trapped inside of the cell in the presence of NIM811, giving the decrease in virus secretion seen in the JFH1 HCVcc infected cells.

In summary, we have found that the lipid trafficking function previously demonstrated for cypA and cyp40 plays an important role in the HCV life cycle [36]. It is likely that lipid trafficking is critical at multiple stages in the virus life cycle, including formation and maintenance of a replication competent membranous web and secretion of the virus. We now show that in addition to inhibition of HCV replication, NIM811 also causes a block in the VLDL pathway resulting in decreased virus release. That cyclophilin inhibitors are now attributed to blocking more than one stage in the virus life cycle lends substantial support for their use in combination with DAAs in the clinic. By targeting multiple stages, these mechanisms may contribute to an enhanced potency of cyclophilin inhibitors, including NIM811 and alisporivir, and by extension increase the barrier to resistance giving infected patients a more favorable outcome [44].

## Materials and methods

### Compounds

NIM811 is a cyclophilin inhibitor with an EC_50 _of 200nM [5]. BILN2061 is an NS3 protease inhibitor with a replicon EC_50 _of 2nM. All compounds were isolated or synthesized at Novartis (Basel, Switzerland).

### Cells

The subgenomic genotype 1b (Con1) HCV replicon cell line, Clone A (designated as sg-1b WT) was obtained from Charles Rice and Apath LLC (St. Louis, Missouri) [49]. NIM811 resistant (NIMr) cells were generated by culturing sg-1b replicon cells in media with increasing concentrations of NIM811, as described previously [5]. Cells were cultured in Dulbecco's modified Eagle's medium supplemented with 2 mM L-glutamine, 0.1 mM nonessential amino acids, and 10% fetal bovine serum (FBS). Replicon cells were maintained in media containing 1 mg/mL geneticin. Huh7.5 cells were used to generate JFH1 infectious virus as described [50].

### Antibodies and Reagents

BODIPY 493/503, DAPI and Alexa Fluor conjugated secondary antibodies for microscopy experiments were purchased from Molecular Probes (Invitrogen). BODIPY 493/503 was dissolved in DMSO at 1 mg/mL and used at 1μg/mL. Rabbit polyclonal antibodies to apoB (ab20737), cyclophilin A (ab41684) and cyclophilin B (ab16045) as well as GAPDH-HRP were obtained from Abcam. Rabbit polyclonal antibody to cyclophilin 40 was from Affinity Bioreagents (PA3-022). Mouse anti-PDI antibody was purchased from Assay Designs (SPA-890). Mouse monoclonal antibody to HCV NS5A was from Meridian Life Sciences (C65388M). ECL anti-rabbit IgG-HRP secondary antibody was from GE Healthcare.

### Indirect Immunofluorescence Microscopy

For confocal microscopy experiments, cells grown in poly-L-lysine coated glass bottom dishes (MatTek Corp.) were fixed with 4% paraformaldehyde for 10 min. followed by permeabilization using 0.01% digitonin dissolved in PBS containing 0.2% gelatin (PBS-G) for 30 minutes. Primary and secondary antibodies were diluted in PBS-G and incubated with the cells for 2 h or 45 min. at room temperature, respectively. After antibody staining, cells were washed with PBS-G and incubated with DAPI and BODIPY 493/503 for 30 minutes. Images were obtained using an Axiovert LSM 510 META laser scanning confocal microscope using a 63X oil immersion objective (Zeiss). Data were processed using LSM 510 Meta software (Zeiss) and images were converted to TIFFs and assembled using ImageJ software (National Institutes of Health, Bethesda, MD).

For quantification of immunofluorescence, cells were grown in 96 well black plates with clear bottoms (Costar). After fixing, permeabilizing and staining (as above), 20X images were acquired and analyzed using a Cellomics ArrayScan VTI High Content Imager and Cellomics ArrayScan software (Thermo Fisher Scientific).

### ApoB ELISA

Sg-1b replicon cells were plated at 1 × 10^4 ^cells/well in a 96 well dish. The next day, media was removed and replaced with complete DMEM containing compound diluted in DMSO (0.5% final concentration of DMSO). After 48h of compound treatment, the supernatants were collected and cleared by centrifugation. ApoB release into the supernatants was quantified using the Human ApoB ELISA kit from AlerCHEK (Portland, ME), Inc according to manufacturer's instructions, with one exception. Supernatants were diluted directly 1:1 with the Apo B specimen diluent provided, with no initial PBS dilution.

### siRNA Transfection

Sg-1b replicon cells were reverse transfected with siGENOME smartpool siRNAs from Dharmacon as described [17]. For confocal microscopy experiments, the transfection mixture and cells were plated in poly-L-lysine coated glass bottom 96 well dishes. Cells were fixed and stained at 96h post-transfection. For quantification, the transfection mixture and cells were plated in black with clear bottom 96 well plates and mean fluorescence intensity (MFI) as well as mean area of lipid droplets was quantified by high content imaging (as above). MFI values are normalized to a RISC-free siRNA control.

### Western Blots

At 72h and 96h post-transfection, sg-1b replicon cells were lysed with RIPA buffer (Sigma Aldrich) containing protease cocktail (Thermo Fisher Scientific). Protein concentrations were determined by BCA Protein Assay (Pierce). Equal amounts of protein mixed with 4X NuPAGE LDS Sample buffer were boiled at 70°C for 10 min., separated by SDS-PAGE, transferred to nitrocellulose by iBlot (Invitrogen) and blocked with 3% bovine serum albumin (BSA) diluted in PBS plus 0.05% Tween-20 (PBST). Primary antibodies were diluted in PBST and incubated with blots overnight at 4°C followed by a 1h incubation with HRP-conjugated secondary antibody. Blots were visualized using ECL (Thermo Fisher Scientific).

### Virus Release Assay

Huh7.5 cells were infected with JFH1 HCVcc for three days to allow the virus to spread throughout the monolayer. Three days post infection, cells were washed 5X with complete DMEM, media was replaced with complete DMEM containing compound in duplicate and incubated at 37°C for 8h. For extracellular virus, one set of supernants was collected and the viral RNA extracted using the QIAamp viral RNA mini kit (Qiagen) according to the manufacturer's instructions, and used to quantify the amount of extracellular viral RNA released. The second set of supernatants was purified using HCV Pure HCV Purification Mini columns (BioVintage) to eliminate any compound from the supernatants. Purified virions were used to determine extracellular infectivity. For intracellular virus, one set of cells was washed with PBS and total RNA extracted using RNeasy RNA Mini kit (Qiagen) according to the manufacturer's instructions. For intracellular infectivity, the second set of cells was trypsinized, pelleted, resuspended in complete DMEM and lysed by four cycles of freeze-thaw (alternating between a dry-ice/ethanol bath and 37°C water bath).

### Infectivity

To measure infectivity of the extracellular and intracellular virus, purified supernatant or lysate was used to infect naïve Huh7.5 cells. At 72hpi, the infectivity read out was measured as renilla luciferase (Promega). Infectivity was normalized to the DMSO control, set to 100%.

### Quantitative Real Time RT-PCR

Copies of HCV RNA in the extracellular supernatant and intracellular lysate were quantified using the Superscript III Platinum One-step Quantitative RT-PCR System (Invitrogen) with the Applied Biosystems 7900HT Real Time PCR System and HCV specific primers and probes. HCV primer sequences: (sense) 5'-CGGGAGAGCCATAGTGG-3' (anti-sense) 5'- AGTACCACAAGGCCTTTCG-3'. Probe: 5'-6FAM-CTGCGGAACCGGTGAGTACAC-TAMRA-3'. Intracellular HCV RNA values were normalized to a GAPDH internal control (TaqMan Assay, Applied Biosystems). Results from four independent experiments, with at least triplicate wells each experiment, were averaged and normalized to the DMSO control.

## Competing interests

The authors declare that they have no competing interests.

## Authors' contributions

LJA designed and executed the experiments, analyzed the data and wrote the manuscript. KL and BW conceived of the studies, assisted in study design and critical discussions of the data. BW participated in manuscript preparation and revisions. TC was involved in revising the manuscript and providing intellectual input. All authors read and approved the final manuscript.
